# Iodine status as indited by neonatal thyroid stimulating hormone screening in Mitre Keluarga Hospital in Surabaya at 2005-2010

**DOI:** 10.1186/1687-9856-2013-S1-P154

**Published:** 2013-10-03

**Authors:** Connie Untario, I Wayan Bikin Suryawan

**Affiliations:** 1 Pediatric Departement of Mitra Keluarga Surabaya Hospital, Indonesia; 2 Pediatric Departement of Wangaya General Hospital,Denpasar, Indonesia

## 

Iodine Deficiency Disorders (IDD) is a significant public health problem in the world. One of the consequences of iodine deficiency is subclinical hypothyroidism during pregnancy and early infancy. Neonatal thyroid screening using serum thyrotropin (TSH) to detect hypothyroidism may also be used to monitor the prevalence of IDD. We aim to evaluate iodine status by neonatal TSH screening in Mitra Keluarga Surabaya Hospital (RSMKS) from year 2005 to 2010.

The study was a cross sectional, hospital-based, and non-interventional in RSMKS conducted from January 2005 to December 2010. Out of the 5619 babies born, 3354 (59.7%) healthy babies with parent’s agreement took part in this study. Blood specimens for TSH measurement were collected between 2 to 6 days after birth and sent to a reference laboraratory. The neonatal TSH values were analysed for iodine deficiency on the basis of WHO/UNICEF/International Council for the Control of IDD criteria.

A total of 3349 newborn babies undergone neonatal TSH screening in RSMKS. The mean of TSH concentration was 5.14 mIU/L. About 1270 (37.9%) had TSH concentration >5mIU/L. There were 166 (27.6%) in 2005, 252 (44.0%) in 2006, 331 (47%) in 2007, 356 (57.5%) in 2008, 114 (20.7%) in 2009 and 51 (16.7%) in 2010 respectively. 22 neonates had TSH >20 mIU/L. Only 2 babies were confirmed positive for hypothyroidism.

Newborn babies in RSMKS from January 2005 to December 2010 is categorised as IDD ranging from mild to severe.This research can be developed further to allow plans for screening of iodine deficiency in pregnant women as well as their newborn babies.

